# One-pot synthesis of poly (3,4-ethylenedioxythiophene)-Pt nanoparticle composite and its application to electrochemical H_2_O_2_ sensor

**DOI:** 10.1186/1556-276X-7-319

**Published:** 2012-06-20

**Authors:** Li-Chi Chang, Huan-Nung Wu, Chia-Yu Lin, Yi-Hsuan Lai, Chih-Wei Hu, Kuo-Chuan Ho

**Affiliations:** 1 Department of Chemical Engineering, National Taiwan University, Taipei, 10617, Taiwan; 2 Institute of Polymer Science and Engineering, National Taiwan University, Taipei, 10617, Taiwan

**Keywords:** H_2_O_2_ electrochemical sensors, Photochemical assisted deposition, Poly (3,4-ethylenedioxythiophene), Pt nanoparticles

## Abstract

Poly(3,4-ethylenedioxythiophene)-Pt nanoparticle composite was synthesized in one-pot fashion using a photo-assisted chemical method, and its electrocatalytic properties toward hydrogen peroxide (H_2_O_2_) was investigated. Under UV irradiation, the rates of the oxidative polymerization of EDOT monomer along with the reduction of Pt^4+^ ions were accelerated. In addition, the morphology of PtNPs was also greatly influenced by the UV irradiation; the size of PtNPs was reduced under UV irradiation, which can be attributed to the faster nucleation rate. The immobilized PtNPs showed excellent electrocatalytic activities towards the electroreduction of hydrogen peroxide. The resultant amperometric sensor showed enhanced sensitivity for the detection of H_2_O_2_ as compared to that without PtNPs, i.e., only with a layer of PEDOT. Amperometric determination of H_2_O_2_ at −0.55 V gave a limit of detection of 1.6 μM (S / N = 3) and a sensitivity of 19.29 mA cm^−2^ M^−1^ up to 6 mM, with a response time (steady state, t_95_) of 30 to 40 s. Energy dispersive X-ray analysis, transmission electron microscopic image, cyclic voltammetry (CV), and scanning electron microscopic images were utilized to characterize the modified electrode. Sensing properties of the modified electrode were studied both by CV and amperometric analysis.

## Background

Research on the quantitative detection of hydrogen peroxide (H_2_O_2_) received considerable attention because H_2_O_2_ is widely used as an oxidizing agent in chemical and food industries [1]. It is an essential mediator in food, pharmaceutical, clinical, and environmental analysis. In addition, H_2_O_2_ is produced during some chemical and enzymatic processes [2,3]; its detection can be used as an indicator for the progress of such processes. Among the developed methodologies [4-7], electrochemical technique is an appropriate alternative or a complementary choice since it has been proved to be an inexpensive and effective way for quantitative determination owing to its intrinsic sensitivity, fast analysis, high selectivity and simplicity. H_2_O_2_ can be detected anodically at a platinum electrode at around +0.7 V vs. SCE; it can also be detected cathodically at a copper electrode at −0.25 V vs. SCE [1].

Many electrode materials, including Pt [8,9], Ag [10], Cu [11], and Prussian blue [12], have been explored as electrocatalysts for the detection of H_2_O_2_. Among these materials, Pt shows excellent electrocatalytic activity towards H_2_O_2_. Recent studies [13-15] show that the improvement in electrocatalytic activity of Pt, in terms of overpotential and sensitivity, can be achieved by the use of the nanosized or nanostructured Pt as compared with its bulk counterpart, owing to its extraordinary surface properties and larger specific surface area. In addition, it has also been reported that the size and shape of Pt play an important role in determining the electrocatalytic activity for H_2_O_2_[16].

The use of conducting polymers as electrode materials in the field of electrocatalysis [17-19] has been a hot research topic not only because they, themselves, exhibit electrocatalytic properties toward many important analytes but also act as effective electrocatalyst support. Regarding the latter, conducting polymers not only can provide sufficient accessible surface area, low resistance, and high stability but also induce uniform distribution of metal nanoparticles and facile electron transfer between electrocatalysts and electrode. Poly(3,4-ethylenedioxythiophene) (PEDOT) has become one of the most intensively studied conducting polymers due to its excellent conductivity, chemical stability, and electrocatalytic properties. In addition to its potential application for the detection of important analytes, such as dopamine [20], nitrite [21], and ascorbic acid [22], the pristine PEDOT and its composite with Pt have also been explored in the fields of fuel cells [23-25], photovoltaics [26-28], and super-capacitors [29,30].

This study reports the preparation of a modified electrode and its application as a sensor for the amperometric detection of H_2_O_2_ based on a screen-printed carbon (SPC) electrode using a composite film of PEDOT and PtNPs designated as PEDOT-PtNPs/SPC electrode. Although there have been reports on the composite film of PtNPs with PEDOT for fuel cell applications [23-25], there is no report on the synthesis of the composite of PEDOT with PtNPs by photo-assisted chemical method and its application for sensing hydrogen peroxide. PEDOT-PtNPs/SPC electrode was prepared firstly by synthesizing PEDOT-PtNP composite in one-pot fashion using a photochemical polymerization method and, subsequently, depositing the composite onto the SPC electrode via the drop-coating method. Cyclic voltammetry (CV) technique was used to study the catalytic reduction of H_2_O_2_ on the PEDOT-PtNPs/SPC electrode. The potential use of the PEDOT-PtNPs/SPCE electrode for the amperometric detection of H_2_O_2_ was discussed.

## Methods

### Chemicals and instruments

3,4-Ethylenedioxythiophene (EDOT, 98%) and chloroplatinic acid hydrate (>99.5%) were purchased from Sigma-Aldrich Corporation (St. Louis, MO, USA) and used as received. Dimethylsulfoxide (DMSO, 99.7%; Sigma-Aldrich) was dehydrated with molecular sieves (4 Å; Acros Organics, New Jersey, USA) before use. H_2_O_2_ sample solution (50 mM) was prepared before each experiment by direct dilution of H_2_O_2_ (35%; Sigma-Aldrich) in deionized water (DIW) and deaerated by purging it with nitrogen for 20 min. Other chemicals were of analytical grade and used without further purification. DIW was used throughout the work.

Electrochemical measurements were carried out using a CHI 440 electrochemical workstation (CH Instruments, Inc., USA) with a conventional three-electrode system; A SPC electrode with a geometric area of 0.071 cm^2^ (Zensor R&D, Taiwan), Ag/AgCl/KCl saturated, and Pt foil are the working electrode, reference electrode, and counter electrode, respectively. All electrochemical experiments were performed at room temperature and all the potentials are reported against the Ag/AgCl/KCl saturated reference electrode.

The nanoscale image of PEDOT-PtNP composite was obtained using scanning electron microscope (SEM, Nova NanoSEM 230, FEI Company, USA); elemental analysis was made using the same SEM with an additional provision of *x*-sight light element energy dispersive X-ray (EDX) spectrometer (6560 INCA, Oxford Instruments, UK). Transmission electron microscopy (TEM, H-7100, Hitachi Ltd., Japan) was also used to obtain the image of PEDOT-PtNP composite. The oxidation state of PtNPs was determined by X-ray photoelectron spectroscopy using an X-ray recorded on a PHI 5000 VersaProbe (ULVAC-PHI, Inc., Chigasaki, Japan) system using a micro-focused (100 μm, 25 W) Al X-ray beam. A Wien-filtered C_60_^+^ ion source (IOG C60-10, Ionoptika Ltd., Chandler's Ford, UK) was operated at 10 nA and 10 kV. The angle between the Ar^+^ and C_60_^+^ ion beam was 33°. The ion-beam current was measured with the target current of a Au foil. The base pressure of the main chamber (<1 × 10^−7^ Pa) was maintained using turbomolecular and ion-getter pumps.

### Preparation of the PEDOT-PtNPs/SPC electrode

**Figure 1:**
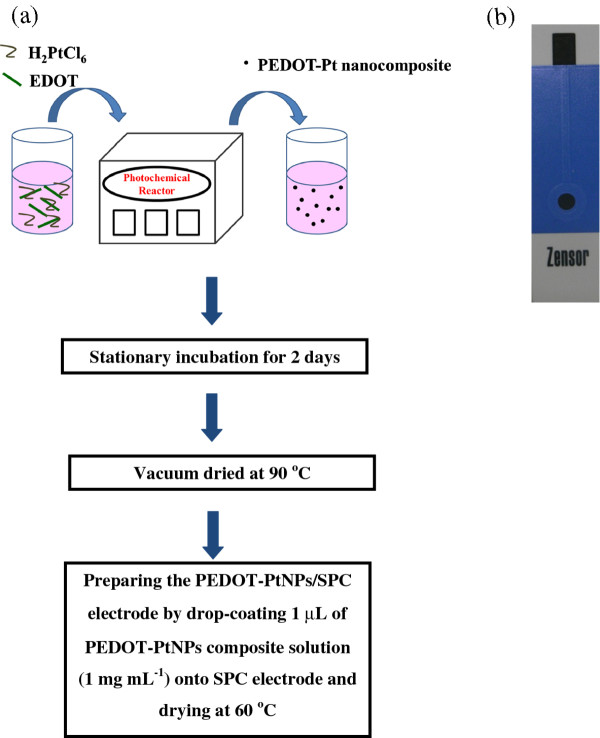
**Scheme for electrode preparation.** (**a**) Flow sheet for the preparation of the PEDOT-PtNPs/SPC electrode. (**b**) The photograph of the SPC electrode.

The synthesis of PEDOT-PtNP composite was carried out by putting a glass bottle, containing 0.01 M of EDOT monomer and 0.001 M of H_2_PtCl_6_ aqueous solution, in a photochemical reactor (Panchum Scientific Corp., Taiwan) followed by irradiation with UV light (power density, 0.14 W cm^−2^; main wavelength, 365 nm) for a specific period of time under forced air convection and mild agitation. As the reaction proceeded, a black-colored suspension was obtained. After UV irradiation, the glass bottle containing black suspension was removed from the photochemical reactor and kept in the dark place at room temperature over 2 days for the precipitation of the composite; after which, the supernatant was removed, and the precipitate was subjected to vacuum dried at 90°C. After being dried, the precipitate was dispersed in DMSO solution at a concentration of 1.0 mg mL^−1^. To prepare the PEDOT-PtNPs/SPC electrode, 1.0 μL of the dispersion was drop-coated onto the SPC electrode, and the coated SPC electrode was dried at 60°C. The scheme of the preparation of PEDOT-PtNPs/SPC electrode is showed in Figure 1.

### Amperometric detection of H_2_O_2_

For the amperometric detection of H_2_O_2_ with amperometry at constant potential by using PEDOT-PtNPs/SPC electrode as the sensor, a suitable sensing potential in the limiting current plateau ranging between 0 and −0.7 V was determined by applying linear sweep voltammetry in a solution containing deaerated 0.1 M PBS (pH 7.4) and 0.5 mM H_2_O_2_ (not shown). Considering the sensitivity and the steadiness of the PEDOT-PtNPs/SPC electrode, the sensing potential was chosen to be −0.6 V. Current densities in the concentration range of 0.4 to 6 mM were collected, and the pertaining calibration curve was constructed for the detection of H_2_O_2_.

## Results and discussion

### Characterization

**Figure 2:**
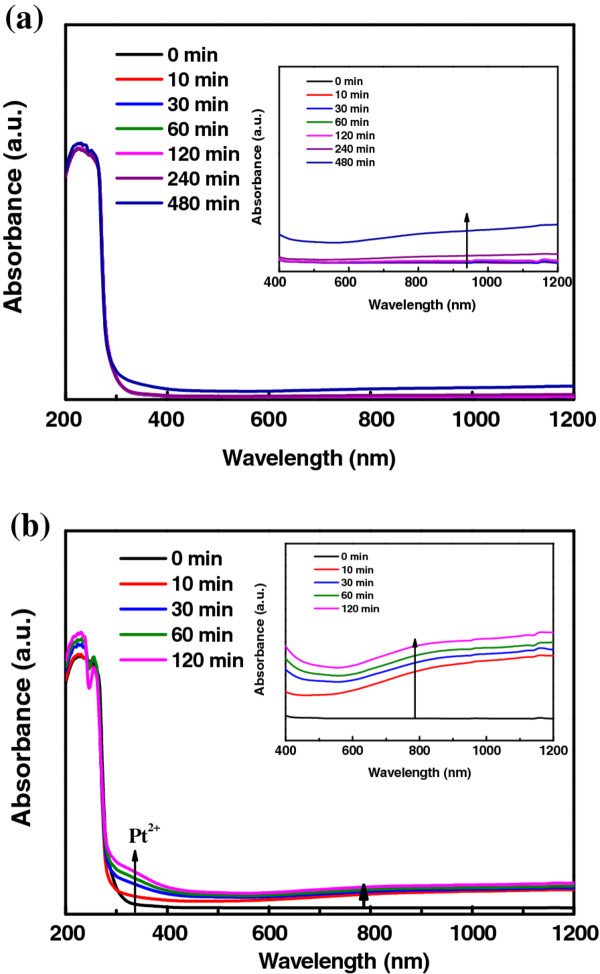
**UV-vis spectra.** UV-vis spectra of the solution containing PEDOT/PtNP composite prepared (**a**) without and (**b**) with UV-irradiation for various periods. The absorbances in the range from 400 to 1200 nm are enlarged for both samples and shown as insets in (**a**) and (**b**).

**Figure 3:**
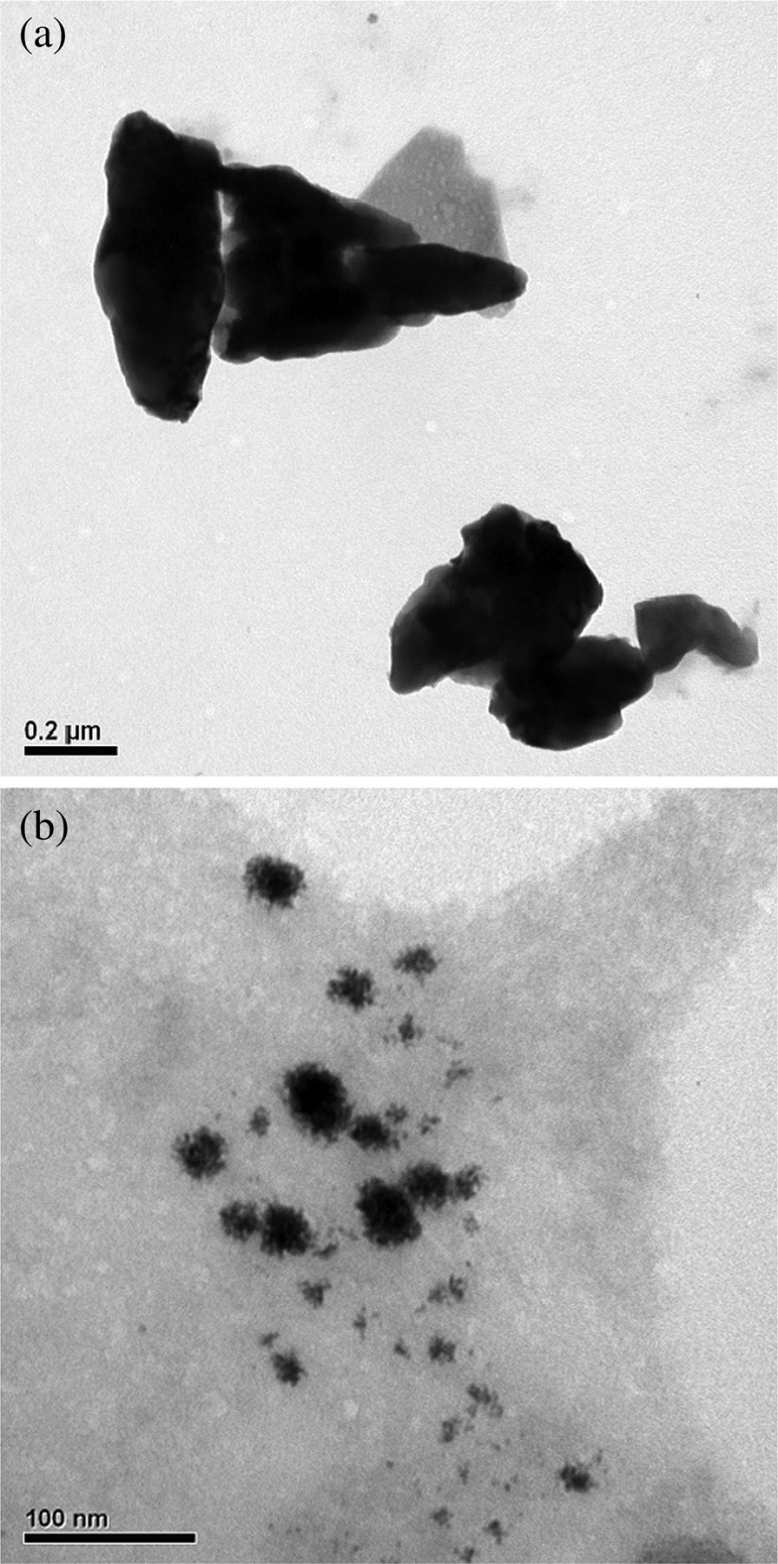
**TEM images.** The TEM images of the PEDOT/PtNP composite synthesized (**a**) without UV irradiation for 480 min and (**b**) synthesized with UV irradiation for 120 min.

Figure 2 shows the UV-vis spectra of the PEDOT-PtNP composite prepared with and without UV irradiation for various periods. The UV-vis spectra were recorded using PEDOT-PtNP composite dispersion prepared by dispersing a 0.1 mL of the reaction solution in the DMSO solvent with the help of sonication. It can be found that the absorbances at wavelengths between 310 and 380 nm, and at wavelengths higher than 700 nm increased monotonically during the time course of the reaction. The increase in the absorbance at wavelengths higher than 700 nm was assigned to the polymerization of EDOT [31]. Therefore, the polymerization rate of EDOT film was catalyzed in presence of UV irradiation as evidenced by observing a faster increase in the absorbance at wavelengths higher than 700 nm. In addition, the increase in absorbance at wavelengths between 310 and 380 nm was assigned to the formation of Pt^2+^[32]. As a result, it was inferred that the polymerization of EDOT was accompanied by the reduction of Pt^4+^ to Pt^2+^, and then to Pt. The kinetics of the polymerization also affected the morphology of the prepared PtNPs. As revealed in Figure 3, PtNPs with smaller size were obtained under UV irradiation, which can be attributed to the faster nucleation rate of PtNPs. 

**Figure 4:**
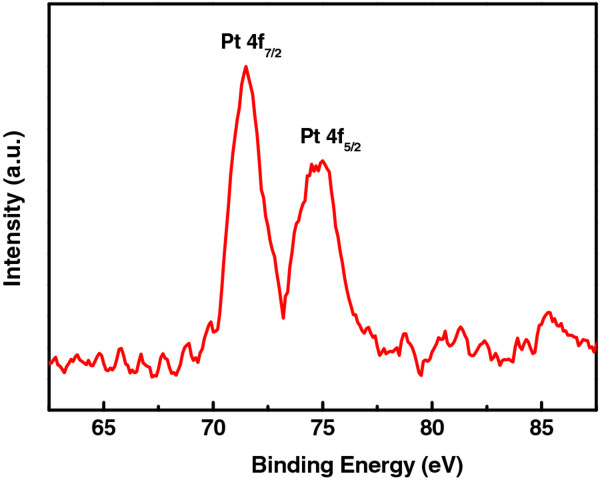
**XPS spectrum.** The Pt 4f XPS spectrum of the PEDOT-PtNP composite synthesized under UV irradiation for 80 min.

The formation of metallic PtNPs from chloroplatinic acid hydrate was further confirmed by XPS analysis. As revealed in Figure 4, the Pt 4f spectrum consists of a spin-split double, i.e., Pt 4f_7/2_ (71.4 eV) and 4f_5/2_ (74.9 eV), which is consistent with the presence of metallic oxidation state of Pt [33]. 

**Figure 5:**
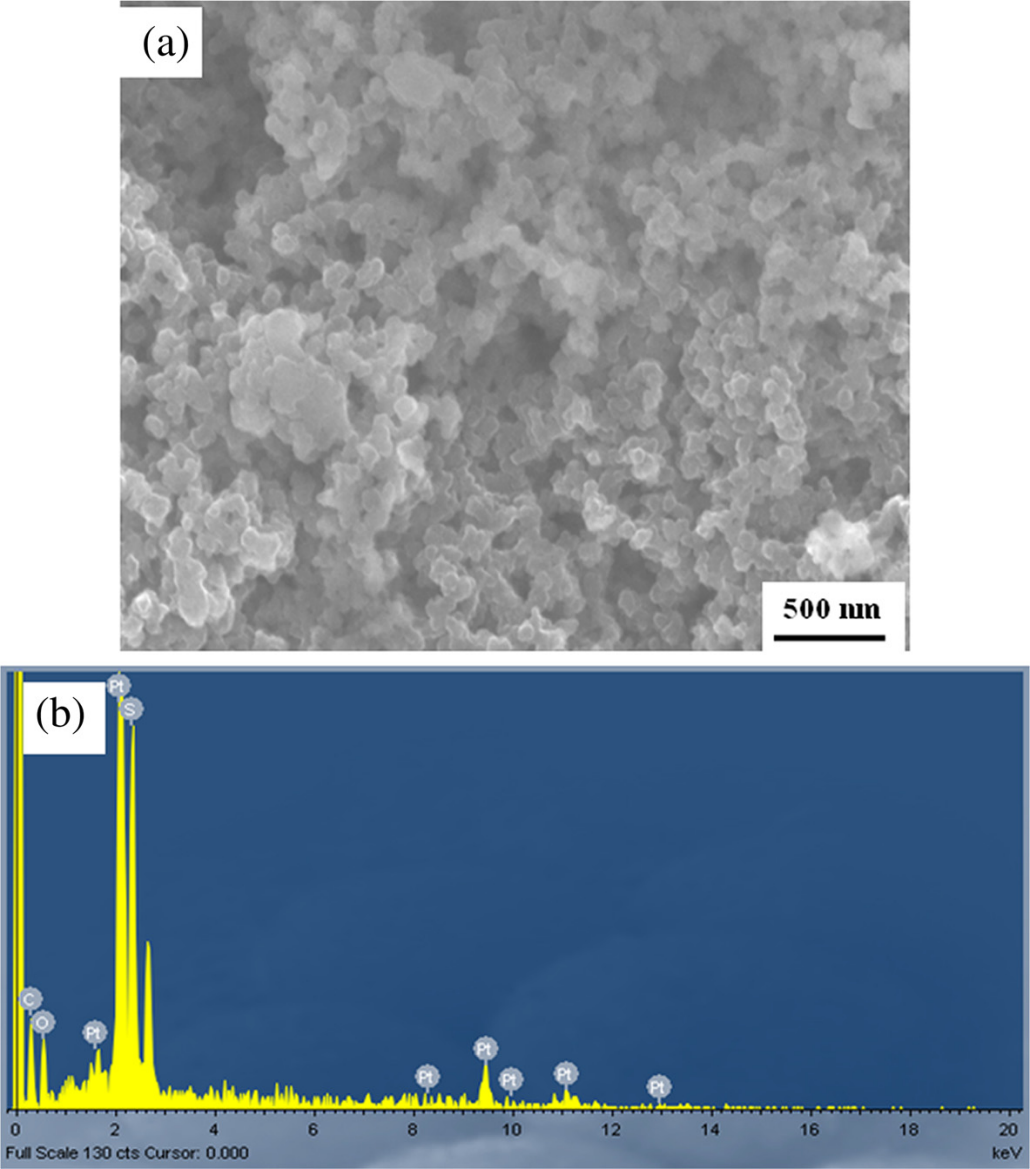
**SEM image and EDX spectrum.** (**a**) SEM image and (**b**) EDX spectrum of the PEDOT-PtNPs/SPC electrode. The PEDOT-PtNP composite was prepared under UV irradiation for 80 min (PEDOT-PtNPs_80 min_).

Figure 5a shows the SEM image of the PEDOT-PtNPs/SPC electrode. As revealed in Figure 5a, the morphology of the PEDOT-PtNP composites is sphere-like, uniform in size (*ca.* 100 nm), and well distributed. In addition, the assembly of nanoparticles creates a 3-day microstructure and nanopores which are beneficial for the diffusion of analytes and would provide highly accessible surface area for the electrocatalytic reaction. Furthermore, from the EDX result, as shown in Figure 5b, the existence of signals for O, S, and Pt confirms that the nanoparticles in Figure 5a belong to PEDOT-PtNP composite.

### Sensing behavior of PEDOT-PtNPs/SPC electrode

**Figure 6:**
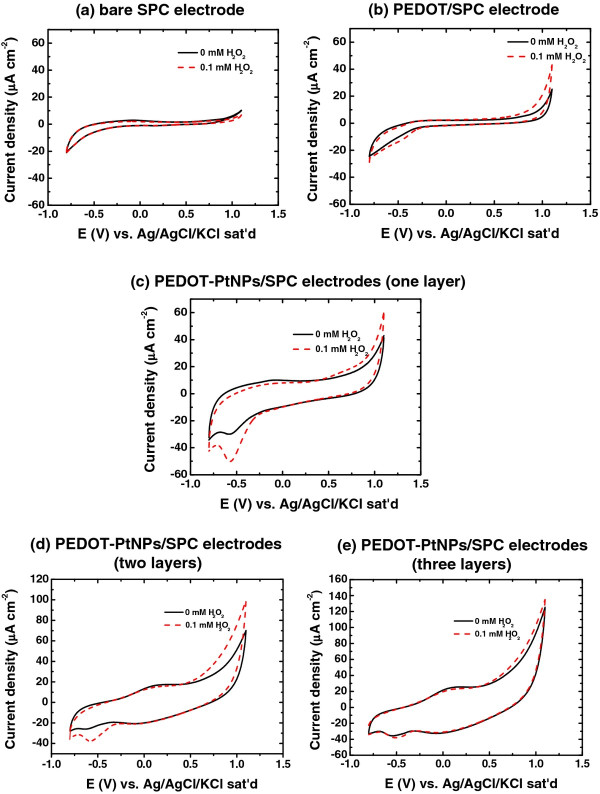
**Cyclic voltammetry.** Cyclic voltammograms of the (**a**) bare SPC, (**b**) PEDOT/SPC, (**c**) PEDOT-PtNPs/SPC electrodes (one layer), (**d**) PEDOT-PtNPs/SPC electrodes (two layers), and (**e**) PEDOT-PtNPs/SPC electrodes (three layers) in 0.1 M phosphate buffer solution (pH 7.4) with and without adding 0.1 mM H_2_O_2_. Scan rate is 25 mV s^−1^.

The electrocatalytic behavior of the PEDOT-PtNPs/SPC electrode towards the electrochemical reduction of H_2_O_2_ was studied using cyclic voltammetry. Figure 6a,b,c shows the CV responses for the bare SPC, PEDOT/SPC, and PEDOT-PtNPs/SPC electrodes in deaerated 0.1 M phosphate buffer solution (PBS, pH 7.4) containing 0 and 0.1 mM of H_2_O_2_. In the blank phosphate buffer, no faradic current was detected for all electrodes. However, an obvious change in reduction current density was noticed after the addition of 0.1 mM of H_2_O_2_ in the case of the PEDOT-PtNPs/SPC electrode, while there were no obvious change in current density for the cases of bare SPC and PEDOT/SPC electrodes. It has been reported that the electroreduction of H_2_O_2_ on PtNPs involves a rate-limiting chemical step (Equation 1) followed by the electron transfer step (Equation 2) [34]:

(1) $${\text{H}}_{2}{\text{O}}_{2}\to {2\text{OH}}_{\text{ads}}^{•}$$

(2) $${\text{OH}}_{\text{ads}}^{•}+e¯\to {\text{OH}}^{-}$$

As a result, we can infer that the enhanced catalytic current of the sensor can mainly be attributed to the presence of the large number of nanosized PtNPs on the electrode [13]. Furthermore, the effect of the film thickness on the sensing performance was also investigated. Here, the film thickness was controlled by adjusting the times of droping-coating. As revealed in Figure 6c,d,e, a higher film thickness reduced the current response to the H_2_O_2_, which could be attributed to a higher diffusion barrier, induced by a thicker film, for the hydroxyl radicals diffuse to the electrode surface.

### Amperometric detection of hydrogen peroxide

**Figure 7:**
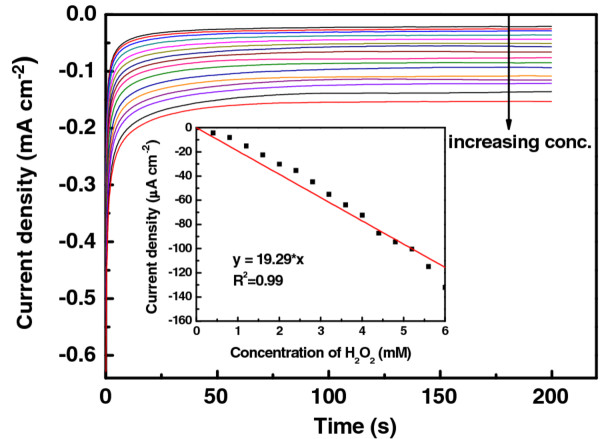
**Amperometric diagram.** The amperometric current responses of the PEDOT-PtNPs/SPC electrode in response to various H_2_O_2_ concentrations, ranging from 0, 0.5, 1.0, 1.5, 2.0, 2.5, 3.0, 3.5, 4.0, 4.5, 5.0, 5.5 to 6 mM, in 0.1 M PBS solution (pH 7.4) at the applied potential of −0.6 V (vs. Ag/AgCl/KCl saturated). The inset shows the calibration curve for H_2_O_2_.

Figure 7 shows the transient current densities at various H_2_O_2_ concentrations. The current density responses of the PEDOT-PtNPs/SPC electrode as a function of the H_2_O_2_ concentration, with a sampling time of 200 s at each concentration level, are measured and shown in Figure 7. The current density increases linearly with the increased H_2_O_2_ concentration up to 6 mM. The sensitivity and detection limit (S / N = 3) for the PEDOT-PtNPs/SPC electrode are 19.29 mA cm^−2^ M^−1^ and of 1.6 μM, respectively.

## Conclusions

The PEDOT-PtNP composite was successfully synthesized in one-pot fashion *via* a novel photochemical method, and its application for the detection of H_2_O_2_ was investigated. The polymerization of EODT accompanied with the formation of PtNPs was confirmed by SEM, TEM, UV-vis, and XPS. In addition, as revealed from the TEM results, the PtNPs were formed and embedded in the nanosized PEDOT, indicating the formation of PEDOT-PtNP composite. As compared with the bare SPC and PEDOT/SPC electrodes, the electrocatalytic activities of PEDOT towards H_2_O_2_ were enhanced by incorporating PtNPs. A linear relationship could be obtained between the current density and the concentration of H_2_O_2_ up to 6 mM, suggesting the successful fabrication of a sensor for the detection of H_2_O_2_ in the concentration range of our interest. The sensitivity of the sensor was determined to be 19.29 mA cm^−2^ M^−1^, and the limit of detection (LOD, with S / N = 3) was 1.6 μM. The response time for reaching steady-state current (t_95_) was 30 to 40 s. Although the conditions for the photochemical reduction of PtNPs were not optimized, the low LOD (approximately 1.6 μM) in this study renders the PEDOT-PtNP electrode attractive for the determination of H_2_O_2_.

## Competing interests

The authors declare that they have no competing interests.

## Author's contributions

Y-HL and C-YL designed the photosynthesis experiments. L-CC and H-NW carried out the photosynthesis experiments. L-CC carried out sensing experiments and drafted the manuscript. SEM and XPS were carried out by C-WH. K-CH supervised the project and was responsible for the accuracy of the data reported. All of the authors discussed and analyzed the data. All authors read and approved the final manuscript.

## Authors' information

LCC received his BS degree in Chemical Engineering from National Taiwan University, Taipei, Taiwan, in 2011. His research interests mainly surround organic-inorganic hybrid materials for chemical sensors. Currently, he is in compulsory military service.

HNW received his BS degree in Chemical Engineering from National Taiwan University, Taipei, Taiwan, in 2011. His research interests include nanomaterials for chemical sensors. He is in compulsory military service now.

CYL received his BS degree in Chemical Engineering from National Cheng Kung University, Tainan, Taiwan, in 2003. He received his MS and PhD degrees in Chemical Engineering from National Taiwan University, Taipei, Taiwan, in 2005 and 2010, respectively. Now, he is a postdoctoral fellow in the Department of Chemistry, University of Cambridge. His research interests mainly surround nanomaterials for chemical sensors and energy related applications.

YHL received her BS degree in Chemical Engineering from National Cheng Kung University, Tainan, Taiwan, in 2007. She received her MS degree in Chemical Engineering from National Taiwan University, Taipei, Taiwan, in 2009. Currently, she is a PhD student in the Department of Chemistry, University of Cambridge. Her research interests focus on dye-sensitized solar cells and photoelectrochemical water splitting.

CWH received his BS and MS degrees in Chemical Engineering from National Chung Cheng University, Chia-Yi, Taiwan, in 2004 and 2006, respectively. He received his PhD degree in the Institute of Polymer Science and Engineering from National Taiwan University, Taipei, Taiwan, in 2011. Now, he is a postdoctoral fellow in the Department of Chemical Engineering at National Taiwan University. His research interests mainly surround conducting polymers for electrochromic, sensors, and solar cells applications.

KCH received BS and MS degrees in Chemical Engineering from National Cheng Kung University, Tainan, Taiwan, in 1978 and 1980, respectively. In 1986, he received the PhD degree in Chemical Engineering at the University of Rochester. That same year, he joined PPG Industries, Inc., first as a Senior Research Engineer and then, from 1990 until 1993, as a Research Project Engineer. He has worked on the electrochemical properties of various electrode materials with emphasis on improving the performances of electrochemical devices, including chemical sensors, electrochromic devices, and dye-sensitized solar cells. Following a six-year industrial career at PPG Industries, Inc., he joined his alma mater at National Cheng Kung University in 1993 as an Associate Professor in the Chemical Engineering Department. In 1994, he moved to the Department of Chemical Engineering at National Taiwan University. Currently, he is a Distinguished Professor jointly appointed by the Department of Chemical Engineering and Institute of Polymer Science and Engineering at National Taiwan University.

## References

[B1] Somasundrum M Kirtikara K Tanticharoen M Dual electrode signal-subtracted biosensor for simultaneous flow injection determination of sucrose and glucose Anal Chim. Acta 1996 319 59 70 10.1016/0003-2670(95)00473-4

[B2] Wang J Lin Y Chen L Organic-phase biosensors for monitoring phenol and hydrogen-peroxide in pharmaceutical antibacterial products Analyst 1993 118 227 280 10.1039/an9931800277 8480909

[B3] Darder M Takada K Pariente F Lorenzo E Abruña HD Dithiobissuccinimidyl propionate as an anchor for assembling peroxidases at electrodes surfaces and its application in a H2O2 biosensor Anal Chem 1999 71 5530 5537 10.1021/ac990759x 10624158

[B4] Hurdis EC Romeyn H Accuracy of determination of hydrogen peroxide by cerate oxidimetry Anal Chem 1954 26 320 325 10.1021/ac60086a016

[B5] Matsubara C Kawamoto N Takamura K Oxo[5,10,15,20-tetra(4-pyridyl)porphyrinato]titanium(iv)—an ultra-high sensitivity spectrophotometric reagent for hydrogen-peroxide Analyst 1992 117 1781 1784 10.1039/an9921701781

[B6] Nakashima K Maki K Kawaguchi S Akiyama S Tsukamoto Y Imai K Peroxyoxalate chemiluminescence assay of hydrogen-peroxide and glucose using 2,4,6,8-tetrathiomorpholinopyrimido[5,4-d]-pyrimidine as a fluorescent component Anal Sci 1991 7 709 719

[B7] Abbas ME Luo W Zhu L Zou J Tang H Fluorometric determination of hydrogen peroxide in milk by using a fenton reaction system Food Chem 2010 120 327 331 10.1016/j.foodchem.2009.10.024

[B8] You TY Niwa O Tomita M Hirono S Characterization of platinum nanoparticle-embedded carbon film electrode and its detection of hydrogen peroxide Anal Chem 2003 75 2080 2085 10.1021/ac026337w 12720344

[B9] Yang MH Yang YH Liu YL Shen GL Yu RQ Platinum nanoparticles-doped sol-gel/carbon nanotubes composite electrochemical sensors and biosensors Biosens Bioelectron 2006 21 1125 1131 10.1016/j.bios.2005.04.009 15885999

[B10] Lin CY Lai YH Balamurugan A Ho KC Electrode modified with a composite film with ZnO nanorods and Ag nanoparticles as a sensor for hydrogen peroxide Talanta 2010 82 340 347 10.1016/j.talanta.2010.04.047 20685476

[B11] Wang Y Wei WZ Zeng JX Liu XY Zeng XD Fabrication of a copper nanoparticles/chitosan/carbon nanotube-modified glassy carbon electrode for electrochemical sensing of hydrogen peroxide and glucose Microchim Acta 2008 160 253 260 10.1007/s00604-007-0844-6

[B12] Ricci F Palleschi G Sensor and biosensor preparation, optimisation and applications of Prussian blue modified electrodes Biosens Bioelecron 2005 21 389 407 10.1016/j.bios.2004.12.001 16076428

[B13] Evans SAG Elliott JM Andrews LM Bartlett PN Doyle PJ Denuault G Detection of hydrogen peroxide at mesoporous platinum microelectrodes Anal Chem 2002 74 1322 1326 10.1021/ac011052p 11924592

[B14] Yang M Qu F Lu Y He Y Shen G Yu R Platinum nanowire nanoelectrode array for the fabrication of biosensors Biomaterials 2006 27 5944 5950 10.1016/j.biomaterials.2006.08.014 16945408

[B15] Hrapovic S Liu Y Male KB Luong JHT Electrochemical biosensing platforms using platinum nanoparticles and carbon nanotubes Anal Chem 2004 76 1083 1088 10.1021/ac035143t 14961742

[B16] Karam P Halaoui LI Sensing of H2O2 at low surface density assemblies of Pt nanoparticles in polyelectrolyte Anal Chem 2008 80 5441 5448 10.1021/ac702358d 18543955

[B17] Jiang Y Wang AY Kan JQ Selective uricase biosensor based on polyaniline synthesized in ionic liquid Sens Actuator B-Chem 2007 124 529 534 10.1016/j.snb.2007.01.016

[B18] Bello A Giannetto M Mori G Seeber R Terzi F Zanardi C Optimization of the DPV potential waveform for determination of ascorbic acid on PEDOT-modified electrodes Sens Actuator B-Chem 2007 121 430 435 10.1016/j.snb.2006.04.066

[B19] Hutchins RS Bachas LG Nitrate-selective electrode developed by electrochemically mediated imprinting doping of polypyrrole Anal Chem 1995 67 1654 1660 10.1021/ac00106a002

[B20] Vasantha VS Chen SM Electrocatalysis and simultaneous detection of dopamine and ascorbic acid using poly(3,4-ethylenedioxy)thiophene film modified electrodes J Electroanal Chem 2006 592 77 87 10.1016/j.jelechem.2006.04.026

[B21] Lin CY Vasantha VS Ho KC Detection of nitrite using poly(3,4-ethylenedioxythiophene) modified SPCEs Sens Actuator B-Chem 2009 140 51 57 10.1016/j.snb.2009.04.047

[B22] Vasantha VS Chen SM Synergistic effect of a catechin-immobilized poly(3,4-ethylenedioxythiophene)-modified electrode on electrocatalysis of NADH in the presence of ascorbic acid and uric acid Electrochim Acta 2006 52 665 674 10.1016/j.electacta.2006.05.052

[B23] Kuo CW Huang LM Wen TC Gopalan A Enhanced electrocatalytic performance for methanol oxidation of a novel Pt-dispersed poly(3,4-ethylenedioxythiophene)-poly (styrene sulfonic acid) electrode J Power Source 2006 160 65 72 10.1016/j.jpowsour.2006.01.100

[B24] Paltras S Munichandraiah N Electrooxidation of methanol on Pt-modified conductive polymer PEDOT Langmuir 2009 25 1732 1738 10.1021/la803099w 19117379

[B25] Drillet JF Dittmeyer R Juettner K Activity and long-term stability of PEDOT as Pt catalyst support for the DMFC anode J Appl Electrochem 2007 37 1219 1226 10.1007/s10800-007-9393-2

[B26] Hong WJ Xu YX Lu GW Li C Shi GQ Transparent grapheme/PEDOT-PSS composite films as counter electrodes of dye-sensitized solar cells Electrochem Commun 2008 10 1555 1558 10.1016/j.elecom.2008.08.007

[B27] Xia JB Masaki N Lira-Cantu M Kim Y Jiang KJ Yanagida S Influence of doped anions on poly(3,4-ethylenedioxythiophene) as hole conductors for iodine-free solid-sate dye-sensitized solar cells J Am Chem Soc 2008 130 1258 1263 10.1021/ja075704o 18171061

[B28] Saito Y Fukuri N Senadeera R Kitamura T Wada Y Yanagida S Solid state dye sensitized solar cells using in situ polymerized PEDOTs as hole conductor Electrochem Commun 2004 6 71 74 10.1016/j.elecom.2003.10.016

[B29] Liu R Lee SB MnO2/poly(3,4-ethylenedioxythiophene) coaxial nanowires by one-step coelectrodeposition for electrochemical energy storage J Am Chem Soc 2008 130 2942 2943 10.1021/ja7112382 18275200

[B30] Lota K Khomenko V Frackowiak E Capacitance properties of poly(3,4-ethylenedioxythiophene)/carbon nanotubes composites J Phys Chem Solids 2004 65 295 301 10.1016/j.jpcs.2003.10.051

[B31] Kumar SS Kumar CS Mathiyarasu J Phani KL Stabilized gold nanoparticles by reduction using 3,4-ethylenedioxythiophene-polystyrenesulfonate in aqueous solutions: nanocomposite formation, stability, and application in catalysis Langmuir 2007 23 3401 3408 10.1021/la063150h 17284059

[B32] Kinyanjui JM Wijeratne NR Hanks J Hatchett DW Chemical and electrochemical synthesis of polyaniline/platinum composites Electrochim Acta 2006 51 2825 2835 10.1016/j.electacta.2005.08.013

[B33] Moulder JF Stickle WF Sobol PE Bomben KD Chastain J Handbook of X-ray photoelectron spectroscopy 1992 2 Physical Electronics Industries, Inc, Eden Prairie, MN 235 236

[B34] Ji S Guo Q Yue Q Wang L Wang L Zhao J Dong R Liu J Jia J Controlled synthesis of Pt nanoparticles array through electroreduction of cisplatin bound at nucleobases terminated surface and application into H2O2 sensing Biosens Bioelectron 2011 26 2067 2073 10.1016/j.bios.2010.09.003 20888213

